# Supplementary material for: Reactive oxygen species-responsive and Raman-traceable hydrogel combining photodynamic and immune therapy for postsurgical cancer treatment
Source: Nat Commun. 2022 Aug 5;13:4553. doi: 10.1038/s41467-022-32160-z (PMC9356008; doi:10.1038/s41467-022-32160-z)
Supplement: Supplementary file 1 — Supplementary Information [file 41467_2022_32160_MOESM1_ESM.pdf]

Supplementary Information

Reactive Oxygen Species-Responsive and Raman-Traceable Hydrogel Combining Photodynamic and Immune Therapy for Postsurgical Cancer Treatment

Yiyi Zhang[1], Sidan Tian[1], Liping Huang[1], Yanan Li[2,5], Yuan Lu[1], Hongyu Li[1], Guiping Chen[6], Fanling Meng[1,3], Gang L. Liu[1], Xiangliang Yang[1,3,4], Jiasheng Tu[2], Chunmeng Sun[2], Liang Luo[1,3,4*]

[1] National Engineering Research Center for Nanomedicine, College of Life Science and Technology, Huazhong University of Science and Technology, Wuhan 430074, China

[2] NMPA Key Laboratory for Research and Evaluation of Pharmaceutical Preparations and Excipients, Department of Pharmaceutics, School of Pharmacy, China Pharmaceutical University, 24 Tong Jia Xiang, Nanjing 210009, China

[3] Key Laboratory of Molecular Biophysics of the Ministry of Education, College of Life Science and Technology, Huazhong University of Science and Technology, Wuhan 430074, China

[4] Hubei Key Laboratory of Bioinorganic Chemistry and Materia Medica, School of Chemistry and Chemical Engineering, Huazhong University of Science and Technology, Wuhan, 430074, China

[5] School of Food Science and Pharmaceutical Engineering, Nanjing Normal University, Nanjing 210023, China

[6] Bruker (Beijing) Scientific Technology Company Limited, Shanghai Branch, Shanghai 200233, China

*Corresponding author: Liang Luo (liangluo@hust.edu.cn)

Supplementary Figures

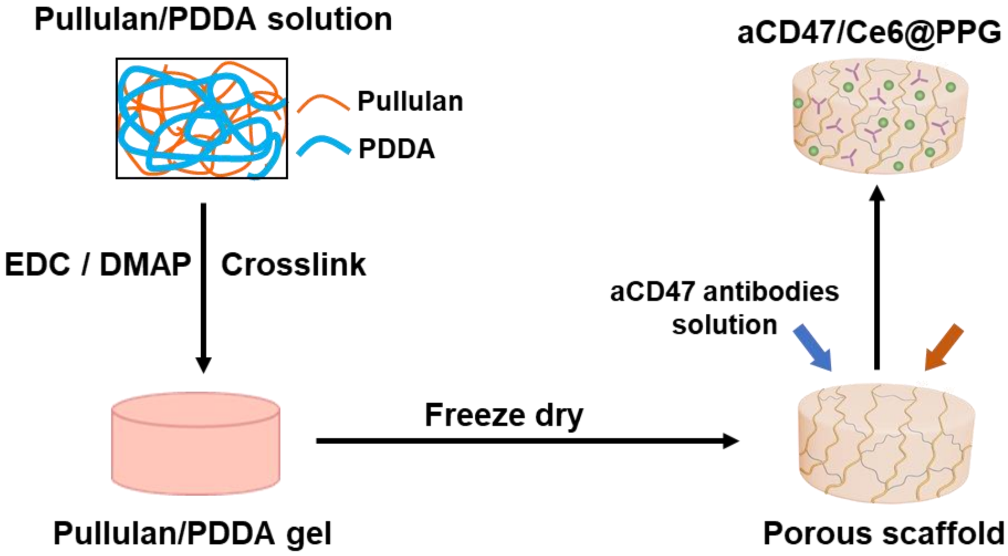

Supplementary Fig. 1 Schematic illustration of the aCD47/Ce6@PPG fabrication process.

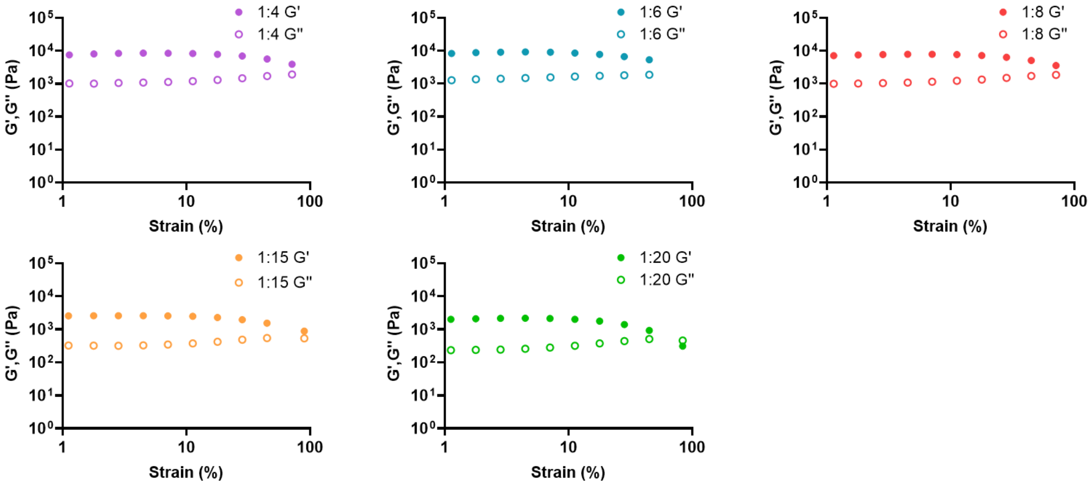

Supplementary Fig. 2 Strain amplitude sweep test of the PPG with different PDDA/Pullulan mass ratios.

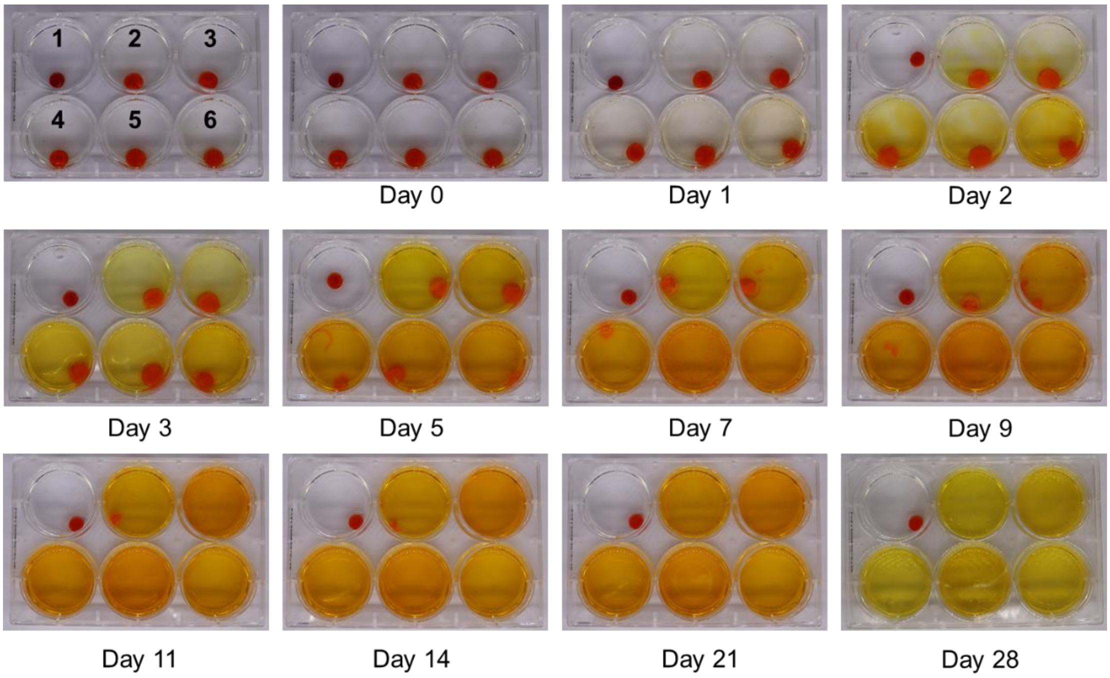

Supplementary Fig. 3 Degradation procedure of PPG at various ROS levels. A PPG hydrogel (PDDA/pullulan mass ratio 1:8) was placed in each well (1–6) containing 1% H_2O_2 solution, which was then added with different amounts of NaClO on a daily basis. The photograph showing the status of PPG was taken at different time after the experiment started. For these wells, 0, 1, 2, 4, 10, 20 μL of NaClO (5%) were added to well 1, 2, 3, 4, 5, and 6 every day, respectively.

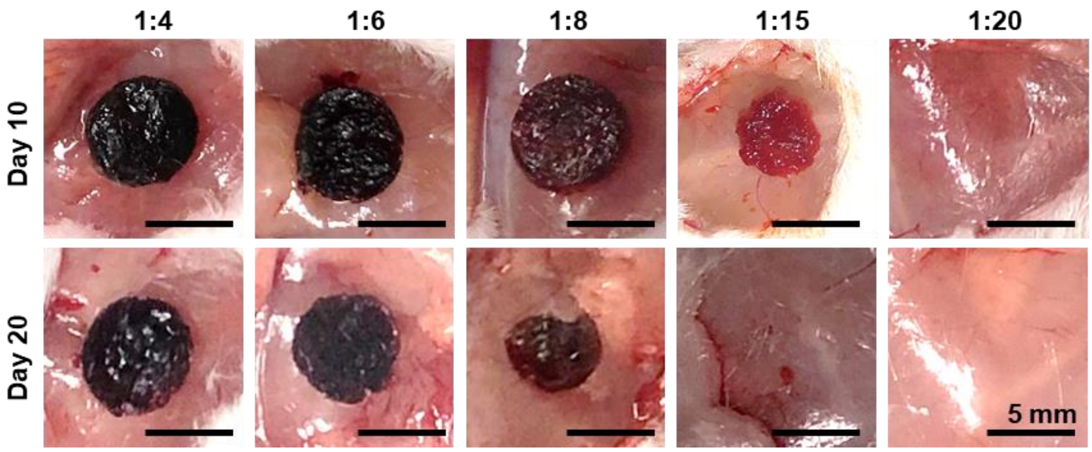

Supplementary Fig. 4 *In vivo* degradation of PPGs on Day 10 and Day 20 post implantation. Scale bar: 5 mm.

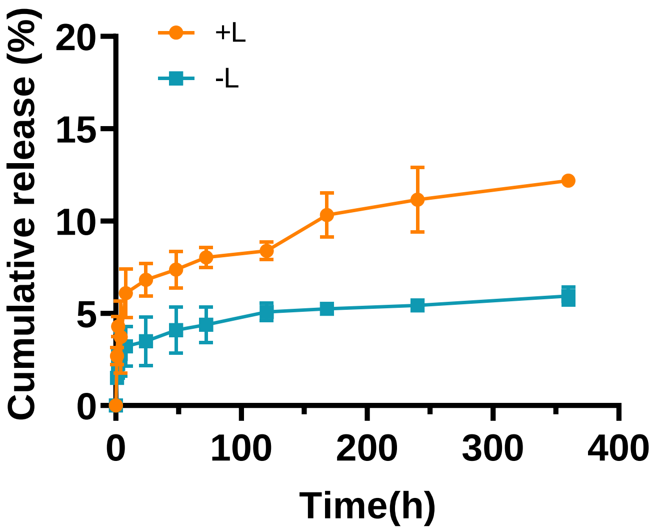

Supplementary Fig. 5 Cumulative release profiles of IgG from IgG/Ce6@PPG with (+L) or without (−L) 640 nm light irradiation. Data are shown as means ± SEM (n = 3 independent samples).

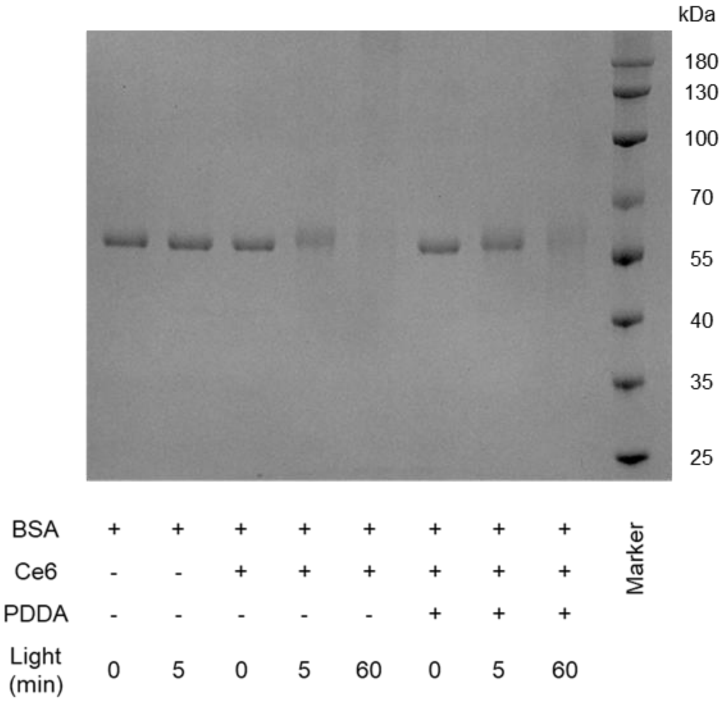

Supplementary Fig. 6 SDS-PAGE analysis of BSA treated in different conditions. BSA 200 μg mL^{-1}; Ce6 10 μg mL^{-1}; PDDA 1 mg mL^{-1}; Light: 640 nm, 5 mW cm^{-2}. This experiment was repeated three times independently with similar results.

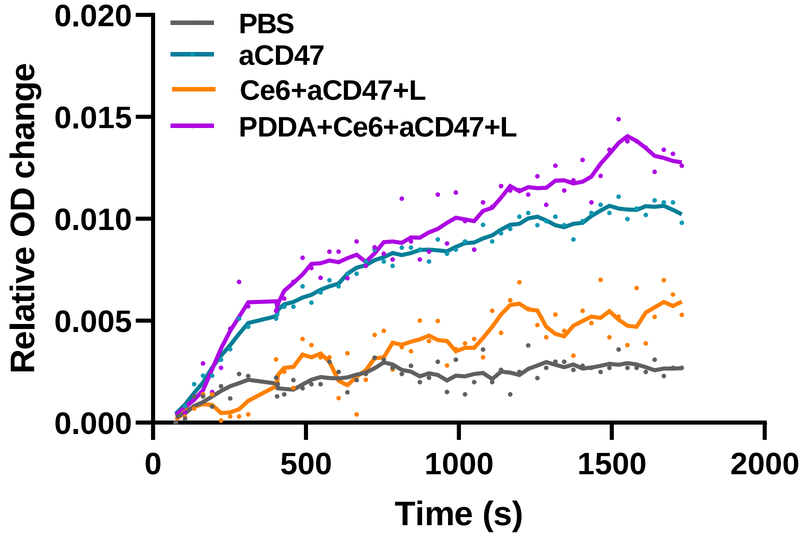

Supplementary Fig. 7 The binding affinity of aCD47 antibody and CD47 antigen measured using the NanoSPR technology under different conditions.

Supplementary Table 1 The binding affinity of aCD47 antibody and CD47 antigen measured using the NanoSPR technology under different conditions.

Samples	K_a ($M^{-1}s^{-1}$)	K_d (s^{-1})	K_D (M)
aCD47+L	8.32×10^{4}	8.47×10^{-5}	1.02×10^{-9}
aCD47+Ce6	4.87×10^{4}	5.30×10^{-5}	1.09×10^{-9}
aCD47+Ce6+L	6.61×10^{4}	3.04×10^{-4}	4.59×10^{-9}
aCD47+Ce6+PPG+L	2.31×10^{4}	5.79×10^{-5}	2.51×10^{-9}

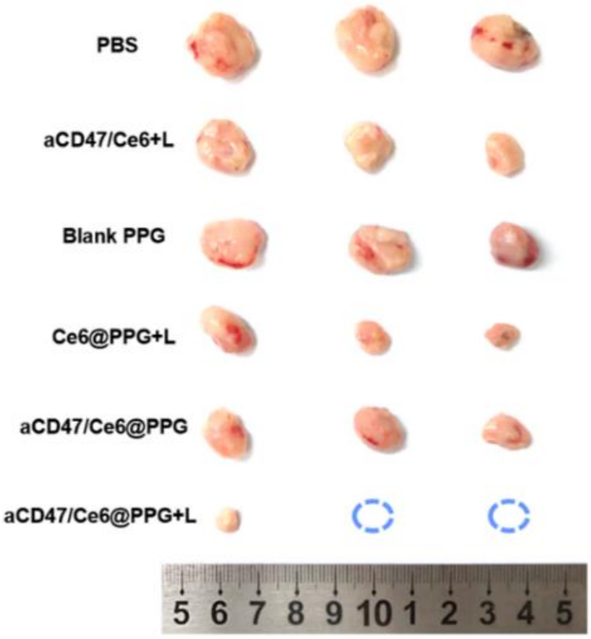

Supplementary Fig. 8 The photograph of recurrent tumors collected from the mice treated in various groups on Day 34 after surgery.

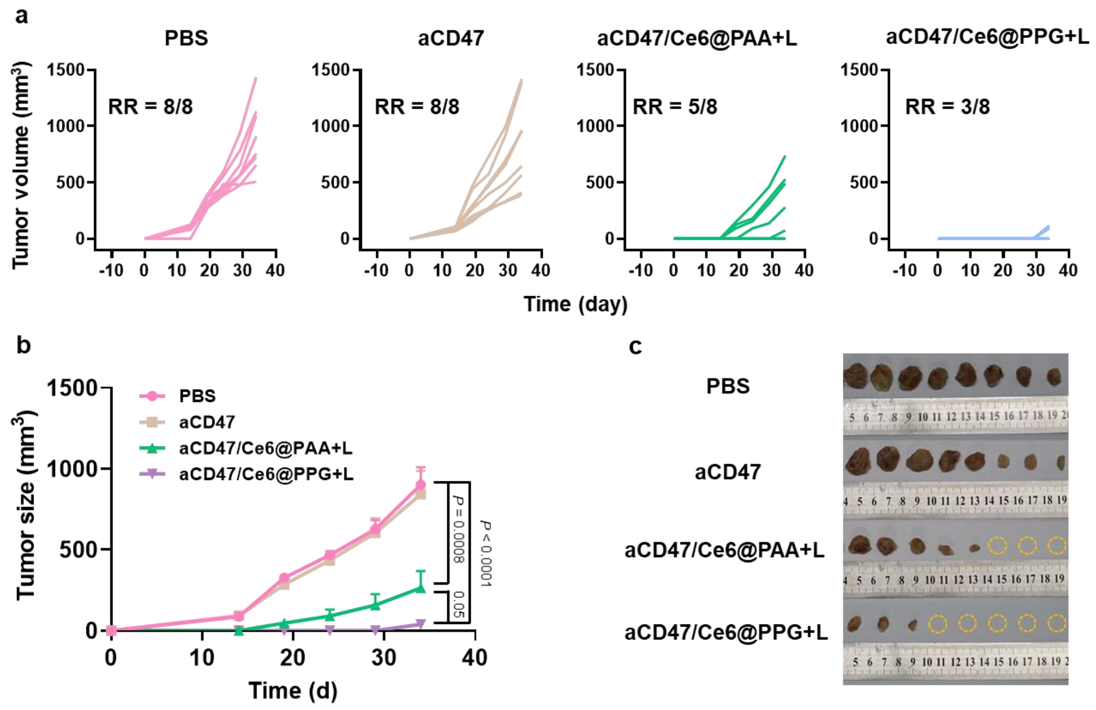

Supplementary Fig. 9 In vivo antitumor effect on 4T1-luc tumor-bearing BALB/c mice by aCD47 solution, aCD47/Ce6@PAA+L, and aCD47/Ce6@PPG+L. **a** Tumor growth curves of individual mouse in different groups. RR: Recurrence rate. **b** Average tumor growth curves of mice treated with PBS, aCD47/Ce6@PAA+L, and aCD47/Ce6@PPG+L. Data are shown as mean ± SEM (n = 8 mice per group). **c** The photograph of recurrent tumors collected from the mice treated in various groups on Day 34 after surgery. Irradiation (640 nm, 5 mW cm^{-2}, 20 min) was applied on Day 0, 2, 4, and 6, respectively. The comparison of two groups was followed by student's t-test (two-tailed). Dose for aCD47: 70 µg per mouse; Dose for Ce6: 80 µg per mouse. * $P < 0.05$, ** $P < 0.01$, and *** $P < 0.001$. Source data and exact P values for b are provided as a Source Data file.

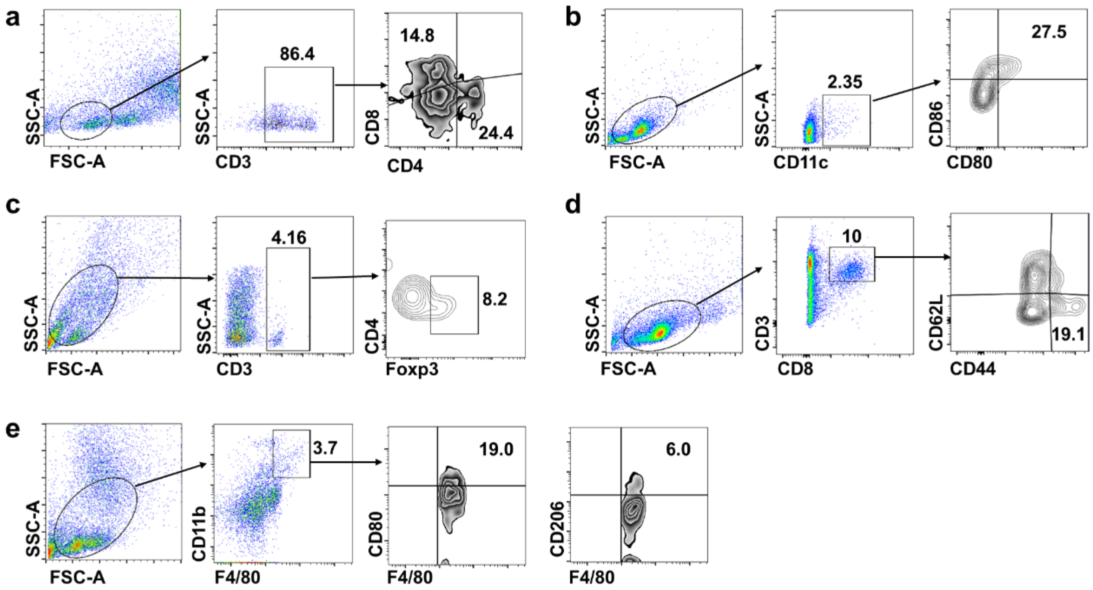

Supplementary Fig. 10 Gating strategies used for cell sorting in flow cytometry analysis. **a** Gating strategy to sort CD8$^+$ (CD3$^+$CD8$^+$) T cells and CD4$^+$ (CD3$^+$CD4$^+$) T cells from Balb/c mice presented on Fig. 5a, 5b, and 5c. **b** Gating strategy to sort matured DC (CD11c$^+$CD80$^+$CD86$^+$) cells from Balb/c mice presented on Fig. 5d. **c** Gating strategy to sort Treg (CD3$^+$CD4$^+$Foxp3$^+$) cells from Balb/c mice presented on Supplementary Fig. 11. **d** Gating strategy to sort T_{EM} (CD3$^+$CD8$^+$CD44$^+$CD62L$^-$) cells from Balb/c mice presented on Fig. 6g. (E) Gating strategy to sort M1-like TAMs (F4/80$^+$CD11c$^+$CD80$^+$) and M2-like TAMs (F4/80$^+$CD11c$^+$CD206$^+$) from Balb/c mice presented on Fig. 5e, 5f and 5g.

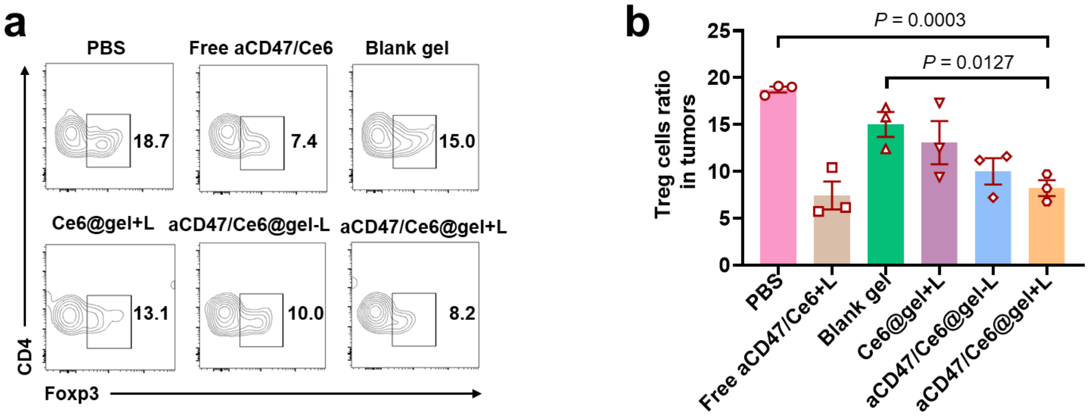

Supplementary Fig. 11 $CD4^+Foxp3^+$ Tregs (gated on $CD3^+CD4^+$ cells) level in recurrent tumors on Day 8 after surgery. **a** Representative flow cytometry analysis. **b** Relative quantification. Data are shown as mean ± SEM (n = 3 mice per group). The statistical comparison between groups was performed following the student's t-test (two-tailed). * $P < 0.05$, ** $P < 0.01$, and *** $P < 0.001$. Source data and exact P values for b are provided as a Source Data file.

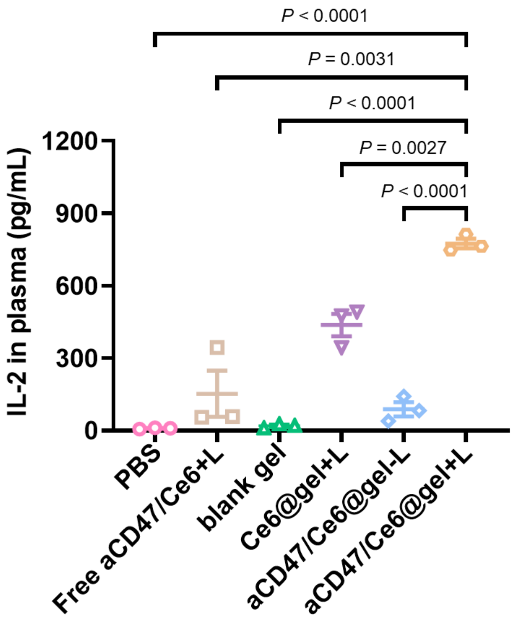

Supplementary Fig. 12 The levels of IL-2 in the serum from mice isolated 8 days after different treatments. Data are presented as mean ± SEM (n = 3 mice per group). The statistical comparison between groups was performed following the student's t-test (two-tailed). * $P < 0.05$, ** $P < 0.01$, and *** $P < 0.001$. Source data and exact P values are provided as a Source Data file.

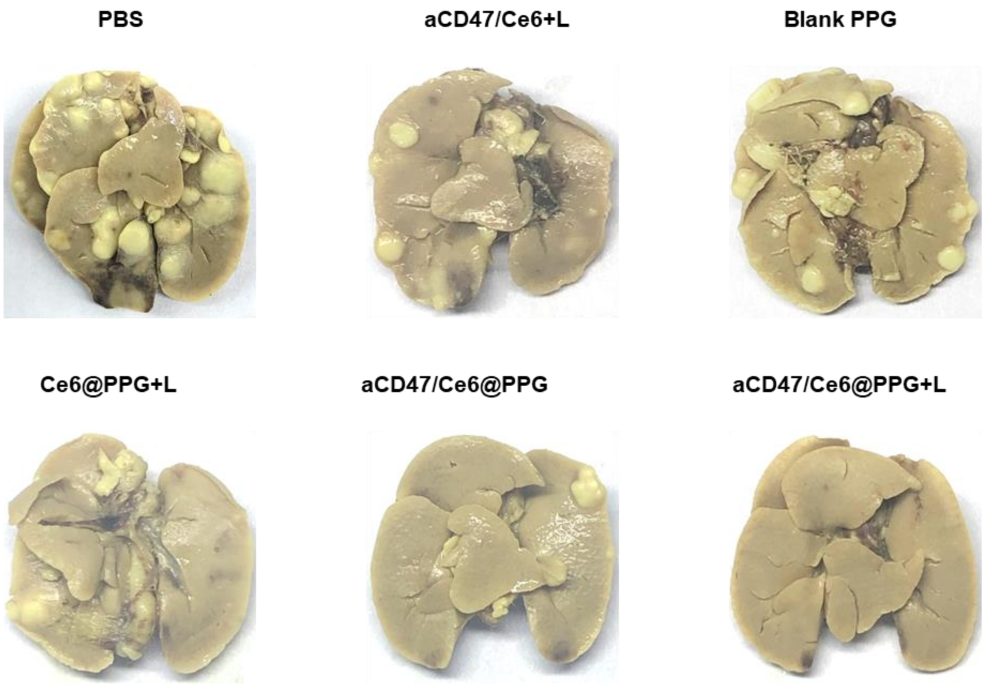

Supplementary Fig. 13 Representative photographs of the back of lungs collected from mice in different groups at day 60 after surgery. Images are representative of 6 biologically independent mice.

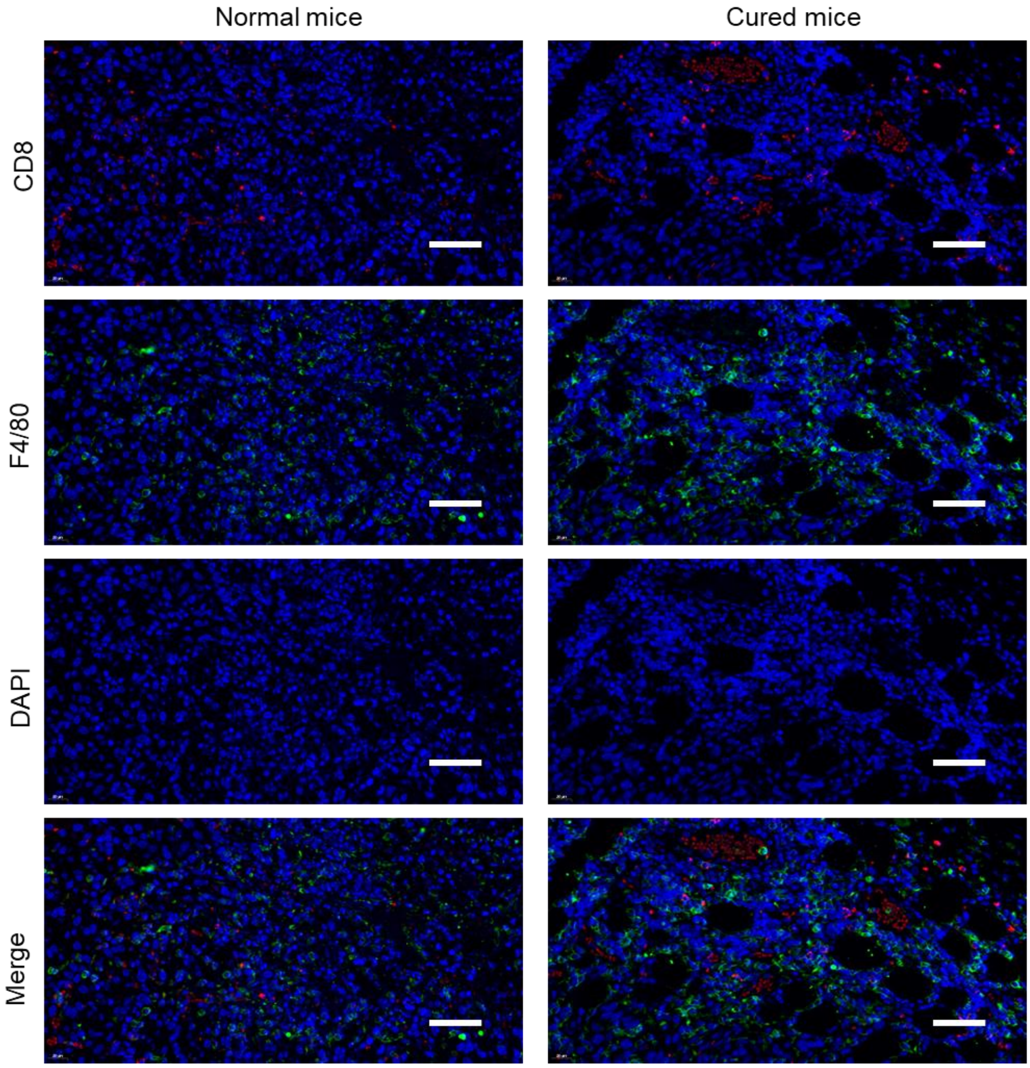

Supplementary Fig. 14 Fluorescence images of $CD8^+$ T cells stained with the Cy3-anti-CD8 antibody (red) and the macrophages were stained with Cy5-anti-F4/80 antibody (green) and the cell nucleus were stained with DAPI (blue). (Scale bar: 50 μm). Images are representative of 2 biologically independent mice.

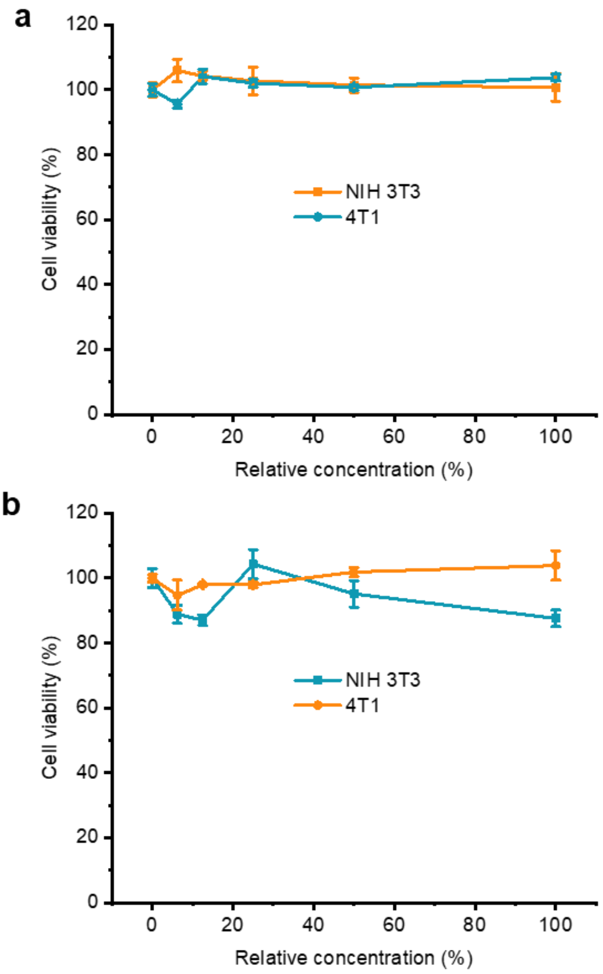

Supplementary Fig. 15 Biocompatibility evaluation of PPG. **a** Viability of NIH3T3 cells and 4T1 cells co-cultured with the leaching solution of blank PPG (diluted by different amounts of DMEM) for 24 h. Data are shown as mean ± SEM (n = 6 biologically independent samples). **b** Viability of NIH3T3 cells and 4T1 cells co-cultured with the leaching solution of PPG (diluted by different amounts of DMEM) for 48 h. Data are shown as mean ± SEM (n = 6 biologically independent samples).

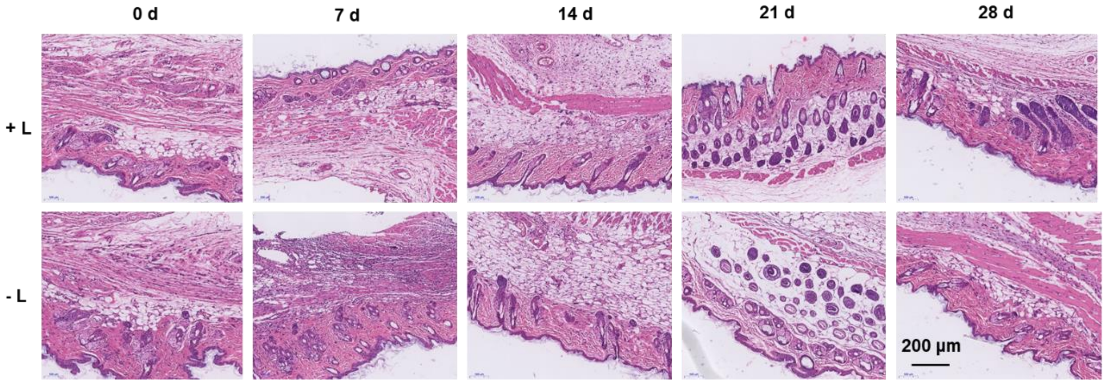

Supplementary Fig. 16 Tissue biocompatibility of the implantable hydrogel with H&E staining of the surrounding skin with (+L) or without (−L) the 640 nm LED irradiation at different testing times (scale bar: 200 μm). Images are representative of 10 biologically independent mice.

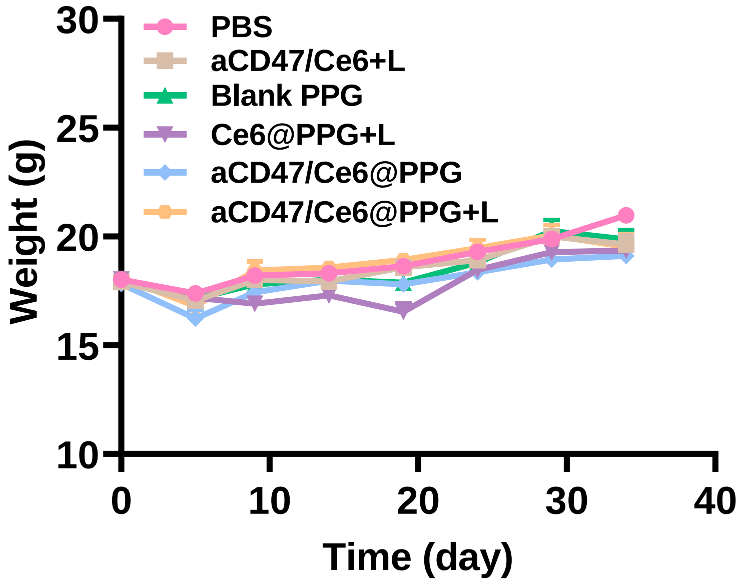

Supplementary Fig. 17 The body weight change curve of 4T1-luc tumor resection mice. Data are shown as mean ± SEM (n = 7–8 mice per group).

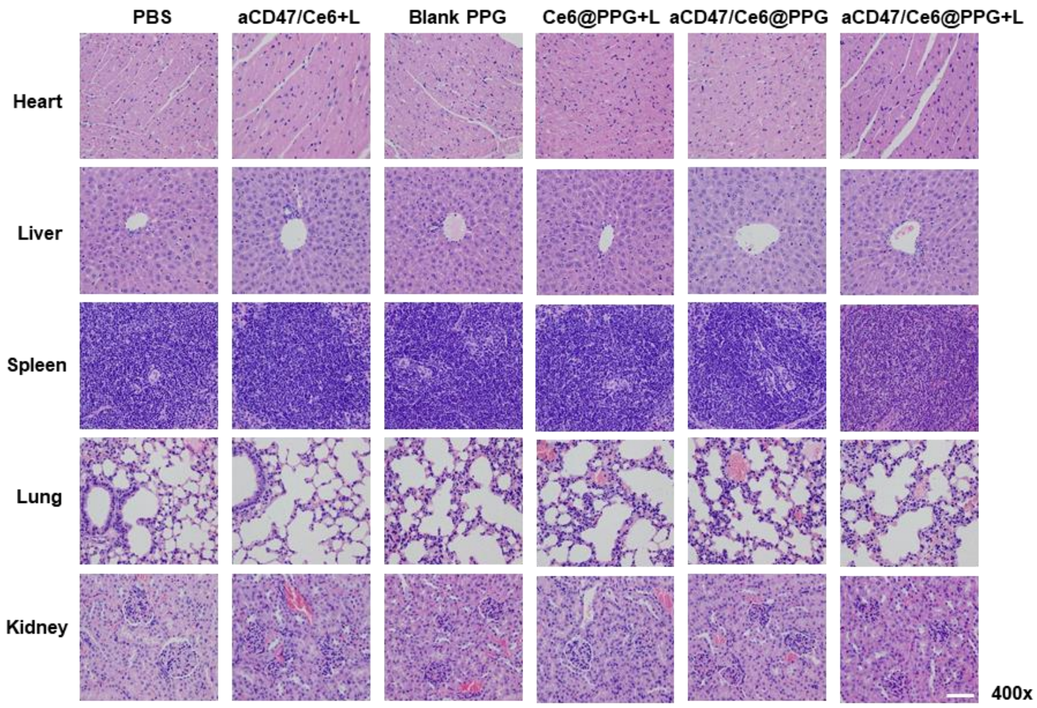

Supplementary Fig. 18 Histopathological images of the main organs, containing heart, liver, spleen, lung, and kidney obtained from the 4T1-luc tumor resection mice. The images were measured at a magnification of 400× (scale bar: 100 μm). Images are representative of 6 biologically independent mice.

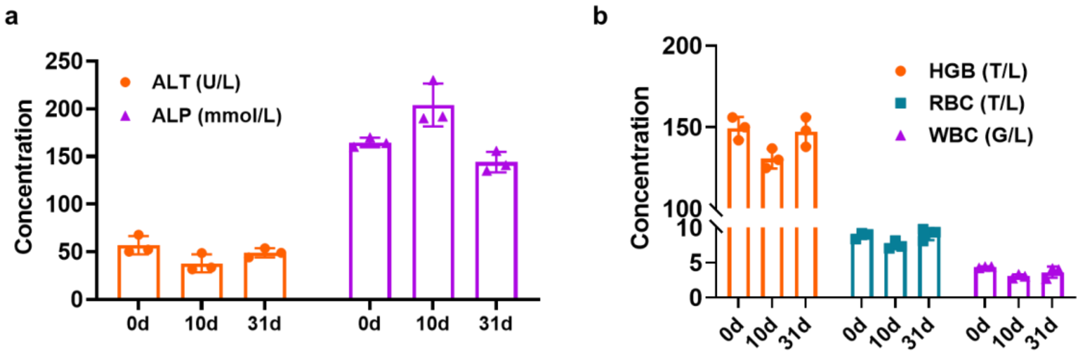

Supplementary Fig. 19 Hemocompatibility of the aCD47/Ce6@PPG. **a** Serum biochemistry assay of mice at different time after receiving aCD47/Ce6@PPG administration. **b** Blood panel test of mice at different time after receiving aCD47/Ce6@PPG administration. Data are shown as mean ± SEM (n = 3 mice per group).
