# Supplementary material for: Reactive oxygen species-responsive and Raman-traceable hydrogel combining photodynamic and immune therapy for postsurgical cancer treatment
Source: Nat Commun. 2022 Aug 5;13:4553. doi: 10.1038/s41467-022-32160-z (PMC9356008; doi:10.1038/s41467-022-32160-z)
Supplement: Supplementary file 2 — Reporting Summary [file 41467_2022_32160_MOESM2_ESM.pdf]

nature portfolio

Corresponding author(s): Liang Luo

Last updated by author(s): Jun 29, 2022

Reporting Summary

Nature Portfolio wishes to improve the reproducibility of the work that we publish. This form provides structure for consistency and transparency in reporting. For further information on Nature Portfolio policies, see our Editorial Policies and the Editorial Policy Checklist.

Statistics

For all statistical analyses, confirm that the following items are present in the figure legend, table legend, main text, or Methods section.

n/a	Confirmed	
☐	☒	The exact sample size (*n*) for each experimental group/condition, given as a discrete number and unit of measurement
☐	☒	A statement on whether measurements were taken from distinct samples or whether the same sample was measured repeatedly
☐	☒	The statistical test(s) used AND whether they are one- or two-sided<br>*Only common tests should be described solely by name; describe more complex techniques in the Methods section.*
☒	☐	A description of all covariates tested
☒	☐	A description of any assumptions or corrections, such as tests of normality and adjustment for multiple comparisons
☐	☒	A full description of the statistical parameters including central tendency (e.g. means) or other basic estimates (e.g. regression coefficient) AND variation (e.g. standard deviation) or associated estimates of uncertainty (e.g. confidence intervals)
☐	☒	For null hypothesis testing, the test statistic (e.g. *F*, *t*, *r*) with confidence intervals, effect sizes, degrees of freedom and *P* value noted<br>*Give P values as exact values whenever suitable.*
☒	☐	For Bayesian analysis, information on the choice of priors and Markov chain Monte Carlo settings
☒	☐	For hierarchical and complex designs, identification of the appropriate level for tests and full reporting of outcomes
☒	☐	Estimates of effect sizes (e.g. Cohen's *d*, Pearson's *r*), indicating how they were calculated

Our web collection on statistics for biologists contains articles on many of the points above.

Software and code

Policy information about availability of computer code

Data collection	Data was collected using IVIS Lumina XR, BD FASCVerse, Kinexus Rotational Rheometer, Bruker SENTERRA II for Raman mapping, Agilent 400-MR DDR2 for NMR spectra, Thermo Fisher Scientific Q Exactive for MS spectra, NGSLabSpec and S&I Vista Control Basic for Raman spectra.
Data analysis	Statistical analyses were performed using GraphPad Prism (v.8.0.2) and OriginLab (2018). FlowJo (V10) was used to process all the flow cytometry data. Living Image (4.3.1) was used to process mouse images. MestRenNova (v.6.1.0-6224) was used to process NMR spectra.

For manuscripts utilizing custom algorithms or software that are central to the research but not yet described in published literature, software must be made available to editors and reviewers. We strongly encourage code deposition in a community repository (e.g. GitHub). See the Nature Portfolio guidelines for submitting code & software for further information.

Data

Policy information about availability of data

All manuscripts must include a data availability statement. This statement should provide the following information, where applicable:

- Accession codes, unique identifiers, or web links for publicly available datasets
- A description of any restrictions on data availability
- For clinical datasets or third party data, please ensure that the statement adheres to our policy

Data Availability. All data generated or analyzed during this study are included in this published article and its Supplementary Information file and the Source Data file.

Field-specific reporting

Please select the one below that is the best fit for your research. If you are not sure, read the appropriate sections before making your selection.

☒ Life sciences ☐ Behavioural & social sciences ☐ Ecological, evolutionary & environmental sciences

For a reference copy of the document with all sections, see nature.com/documents/nr-reporting-summary-flat.pdf

Life sciences study design

All studies must disclose on these points even when the disclosure is negative.

Sample size	Details regarding the sample size of all experiments are provided in Methods section and figure legends. Sample sizes were based on our previous experience and other publications (Nature Communications 2019, 10, 4871; Nature Communications 2020, 11, 1857). Reasonable sample sizes were chosen to ensure they are sufficient for statistical comparison between different groups.
Data exclusions	No data were excluded from the analyses.
Replication	In vitro experiments were completed in duplicate or triplicate to successfully verify reproducibility. In vivo experiments were completed in triplicate as noted by up to three people to successfully verify reproducibility and the results were pooled as noted.
Randomization	For in vitro test, samples were randomly allocated to corresponding experimental groups. For in vivo test, mice were inoculated tumor at the same time and then randomly assigned to a group for similar average tumor sizes.
Blinding	The investigators were blinded to the group allocation during data collection and analysis, the tumor size measurement, tissue harvesting and processing.

Reporting for specific materials, systems and methods

We require information from authors about some types of materials, experimental systems and methods used in many studies. Here, indicate whether each material, system or method listed is relevant to your study. If you are not sure if a list item applies to your research, read the appropriate section before selecting a response.

Materials & experimental systems

n/a	Involved in the study	
☐	☒	Antibodies
☐	☒	Eukaryotic cell lines
☒	☐	Palaeontology and archaeology
☐	☒	Animals and other organisms
☒	☐	Human research participants
☒	☐	Clinical data
☒	☐	Dual use research of concern

Methods

n/a	Involved in the study	
☒	☐	ChIP-seq
☐	☒	Flow cytometry
☒	☐	MRI-based neuroimaging

Antibodies

Antibodies used

Anti-mouse CD47 antibodies (BioX Cell, Catalog no. BE0270, clone MIAP301, without dilution or 1/6 dilution)
AF790-labeled goat anti-mouse IgG (H&L) (Jackson ImmunoResear, Catalog no.115-655-146, RRID AB_2338944)
Anti-mouse CD3-PerCP-Cy5.5 (BD Biosciences, Catalog no. 551163, clone 145-2C11, 1/50 dilution)
Anti-mouse CD4-APC (BD Biosciences, Catalog no.553051, clone RM4-5, 1/200 dilution)
Anti-mouse CD8-FITC (BD Biosciences, Catalog no. 553030, clone 53-6.7, 1/200 dilution)
Anti-mouse CD86-PE (BD Biosciences, Catalog no. 553692, clone GL1, 1/200 dilution)
Anti-mouse CD80-APC (BD Biosciences, Catalog no. 560016, clone 16-10A1, 1/200 dilution)
Anti-mouse Foxp3-PE (BD Biosciences, Catalog no. 563101, clone R16-715, 1/200 dilution)
Anti-mouse CD11c-FITC (BD Biosciences, Catalog no. 557400, clone HL3, 1/200 dilution)
Anti-mouse CD62L-BV421 (BD Biosciences, Catalog no. 562910, clone MEL-14, 1/200 dilution)
Anti-mouse CD44-APC (BD Biosciences, Catalog no. 559250, clone IM7, 1/200 dilution)
Anti-mouse CD11b-PE-Cy7 (BD Biosciences, Catalog no.552850, clone M1/70, 1/200 dilution)
Anti-mouse CD206-PE (Biolegend, Catalog no. 141705, clone C068C2, 1/200 dilution)
Anti-mouse F4/80-FITC (Biolegend, Catalog no. 123107, clone BM8, 1/200 dilution)

Validation

All antibodies were validated by manufacturers, with related data shown on the manufacturer website. Additional validation of both therapeutic and flow cytometric antibodies was not performed by the authors. All validation statements can be found on the respective antibody website:

Anti-mouse CD47 antibodies: https://bxcell.com/product/invivomab-mouse-cd47-iap/
AF790-labeled goat anti-mouse IgG (H&L): https://www.jacksonimmuno.com/catalog/products/115-655-146
Anti-mouse CD3-PerCP-Cy5.5: https://www.bdbiosciences.com/en-eu/products/reagents/flow-cytometry-reagents/research-reagents/single-color-antibodies-ruo/percp-cy-5-5-hamster-anti-mouse-cd3e.551163
Anti-mouse CD4-APC: https://www.bdbiosciences.com/en-eu/products/reagents/flow-cytometry-reagents/research-reagents/single-color-antibodies-ruo/apc-rat-anti-mouse-cd4.553051
Anti-mouse CD8-FITC: https://www.bdbiosciences.com/en-eu/products/reagents/flow-cytometry-reagents/research-reagents/single-color-antibodies-ruo/fitc-rat-anti-mouse-cd8a.553030
Anti-mouse CD86-PE: https://www.bdbiosciences.com/en-eu/products/reagents/flow-cytometry-reagents/research-reagents/single-color-antibodies-ruo/pe-rat-anti-mouse-cd86.553692
Anti-mouse CD80-APC: https://www.bdbiosciences.com/en-eu/products/reagents/flow-cytometry-reagents/research-reagents/single-color-antibodies-ruo/apc-hamster-anti-mouse-cd80.560016
Anti-mouse Foxp3-PE: https://www.bdbiosciences.com/en-eu/products/reagents/flow-cytometry-reagents/research-reagents/single-color-antibodies-ruo/pe-rat-anti-mouse-foxp3.563101
Anti-mouse CD11c-FITC: https://www.bdbiosciences.com/en-eu/products/reagents/flow-cytometry-reagents/research-reagents/single-color-antibodies-ruo/fitc-hamster-anti-mouse-cd11c.557400
Anti-mouse CD62L-BV421: https://www.bdbiosciences.com/en-eu/products/reagents/flow-cytometry-reagents/research-reagents/single-color-antibodies-ruo/bv421-rat-anti-mouse-cd62l.562910
Anti-mouse CD44-APC: https://www.bdbiosciences.com/en-eu/products/reagents/flow-cytometry-reagents/research-reagents/single-color-antibodies-ruo/apc-rat-anti-mouse-cd44.559250
Anti-mouse CD11b-PE-Cy7: https://www.bdbiosciences.com/en-eu/products/reagents/flow-cytometry-reagents/research-reagents/single-color-antibodies-ruo/pe-cy-7-rat-anti-cd11b.552850
Anti-mouse CD206-PE: https://www.biolegend.com/en-us/products/pe-anti-mouse-cd206-mmr-antibody-7424?GroupID=BLG9506
Anti-mouse F4/80-FITC: https://www.biolegend.com/en-us/products/fitc-anti-mouse-f4-80-antibody-4067?GroupID=BLG5319

Eukaryotic cell lines

Policy information about cell lines

Cell line source(s)	The metastatic murine 4T1-luc breast cancer cells and the fibroblast NIH 3T3 cells were purchased from the American Type Culture Collection (ATCC).
Authentication	The cell lines were morphologically confirmed according to the information provided by ATCC.
Mycoplasma contamination	All cell lines showed negative for mycoplasma contamination.
Commonly misidentified lines (See ICLAC register)	No commonly misidentified cell lines were used in this study.

Animals and other organisms

Policy information about studies involving animals; ARRIVE guidelines recommended for reporting animal research

Laboratory animals	BALB/c mice (6–8 weeks old, 18–20 g) were purchased from the Liaoning Changsheng Biotechnology Co., Ltd. (Benxi, China) and the Qinglongshan Farms (Nanjing, China). All animals were bred in a pathogen-free facility with a 12 h light/dark cycle at 20 ± 3 °C and relative humidity of 40%-70%, and had ad libitum access to food and water. Animal protocols were performed under the guidelines for human and responsible use of animals in research set by Huazhong University of Science and Technology and China Pharmaceutical University.
Wild animals	This study did not involve wild animals.
Field-collected samples	This study did not involve field-collected samples.
Ethics oversight	Animal protocols were performed according to protocols approved by the Institutional Animal Care and Use Committees of Huazhong University of Science and Technology (IACUC Number: 2832) and China Pharmaceutical University (2019-08-003).

Note that full information on the approval of the study protocol must also be provided in the manuscript.

Flow Cytometry

Plots

Confirm that:

- [x] The axis labels state the marker and fluorochrome used (e.g. CD4-FITC).
- [x] The axis scales are clearly visible. Include numbers along axes only for bottom left plot of group (a 'group' is an analysis of identical markers).
- [x] All plots are contour plots with outliers or pseudocolor plots.
- [x] A numerical value for number of cells or percentage (with statistics) is provided.

Methodology

Sample preparation	Tumors, spleen, and tumor-draining lymph nodes were harvested from sacrificed mice. The tumors and lymph nodes were cut into small pieces and resuspended in collagenase D in DMEM (1 mg/mL). The solutions were incubated for 1 h at 37 °C on a shaker (90 rpm) and filtered through a 70-µm Falcon cell strainer. The supernatant from the digested tumor tissues was collected, centrifuged at 490 g for 5 min, and resuspended. The spleen was mechanically dissociated and resuspended in DMEM. The suspension was filtered through a 70-µm Falcon cell strainer, centrifuged and resuspended. Erythrocytes were lysed with red blood cell lysis buffer for 5 min at 37 °C. Cell suspensions were prepared as described above and then stained with the antibodies. The cells were then washed twice and analyzed using flow cytometer.
Instrument	BD FASCVerse Flow Cytometry
Software	Flowjo_V10
Cell population abundance	The cells were extracted by specific gravity separation methods with 70% percoll or 40% percoll.
Gating strategy	Gating was first based on FSC/SSC and singlet cells were gated for further analysis. The cell populations were then analyzed based on expression of markers. Gating was then based on positive level.

☒ Tick this box to confirm that a figure exemplifying the gating strategy is provided in the Supplementary Information.
